# Depressiveness, symptoms of anxiety and cognitive dysfunctions in patients with asthma and chronic obstructive pulmonary disease (COPD): possible associations with inflammation markers: a pilot study

**DOI:** 10.1007/s00702-014-1171-9

**Published:** 2014-02-15

**Authors:** Agnieszka Bratek, Karolina Zawada, Julia Beil-Gawełczyk, Sonia Beil, Ewa Sozańska, Krzysztof Krysta, Adam Barczyk, Irena Krupka-Matuszczyk, Władysław Pierzchała

**Affiliations:** 1 Department of Psychiatry and Psychotherapy, Independent Public Clinical Hospital No. 7 of Medical University of Silesia, Ziołowa 45, Katowice, 40-635 Poland; 2 Department of Pulmonology, Central Clinical Hospital of Medical University of Silesia, Katowice, Poland; 3 Provincial Specialist Hospital No. 3, Rybnik, Poland

**Keywords:** Anxiety, Asthma, Comorbidity, COPD, Depression, Psychiatry, Psychosomatics, Pulmonology

## Abstract

Psychiatric symptoms of anxiety, depression and cognitive dysfunction often occur in patients suffering from somatic conditions such as asthma and chronic obstructive pulmonary disease (COPD) which constitute a major and growing public health problem. In the present study we therefore aimed at analyzing depressive symptoms as well as symptoms of anxiety and cognitive problems in patients with mild to moderate asthma and COPD. 59 participants—17 with asthma, 24 with COPD and 18 healthy controls were enrolled. Depressiveness was assessed with the beck depression inventory (BDI); anxiety symptoms were measured with the State-Trait Anxiety Inventory Part 1 and 2, and cognitive function levels were estimated with the Trail Making Test Part A and B. A score above the threshold indicative for depression was found by 33 % (*n* = 8) of COPD patients, 29 % (*n* = 5) of asthma patients compared to 0.05 % (*n* = 1) of the control group. Clinically relevant anxiety levels were found in 42 % (*n* = 10) of the COPD group, 41 % (*n* = 7) of the asthma patients and 17 % (*n* = 3) of the controls. Patients with COPD performed significantly worse on the TMT than other groups. Psychoemotional state and cognitive functions were found to be correlated with exposure to tobacco smoke (measured in pack-years) and airway obstruction (measured with FEV1). In conclusion, patients with mild to moderate asthma and COPD exhibit significantly higher levels of depressive and anxiety symptoms as well as cognitive dysfunctions than controls. The prevalence of these symptoms is related to the amount of exposure to tobacco smoke and the severity of airflow obstruction.

## Introduction

Psychiatric disorders occur quite frequently in the general population. The most frequent psychiatric disorders are anxiety disorders (14 %) and mood disorders (7.8 %) (Wittchen et al. 2011). In addition, psychiatric comorbidities are often observed in patients suffering from chronic somatic diseases. Especially high prevalence rates of cognitive dysfunctions, depression and anxiety disorders have been reported for patients suffering from asthma and COPD (van Ede et al. 1999; Maurer et al. 2008; Asnaashari et al. 2012). These psychiatric comorbidities have a significant negative impact on a patient’s quality of life. In one study, the Asthma Related Quality of Life (AQOL) scores were negatively associated with depression. In addition, greater anxiety was associated with an increased perception of asthma-specific panic-fear and hyperventilation symptoms during an asthma attack, irrespective of the depression status (Deshmukh et al. 2008). Higher anxiety and depression levels were also associated with greater levels of fatigue, shortness of breath and frequency of COPD symptoms (Doyle et al. 2013). In another study evaluating the relative impact of having depressive and/or anxiety disorder on asthma control and quality of life, both depression and anxiety were associated with worse quality of life, and depression, but not anxiety disorder, was additionally associated with worse asthma control (Lavoie et al. 2006).

Apart from a reduced quality of life, there are also socioeconomic implications: For example, it was reported that patients with psychiatric comorbidities were spending twice as long time in hospital (Yellowlees et al. 1987). A more recent study confirms the hypothesis that adults with both mental disorders and asthma have significantly higher rates of functional impairment and use of mental health services, compared with those with either asthma or mental disorders alone (Goodwin et al. 2010).

Many authors draw their attention to the connection between the mental state of patients, progression of the disease and the effectiveness of treatment (Rietveld and Creer 2003; Lavoie et al. 2005; Trzcińska et al. 2012). Patients with cognitive deficits follow doctors’ recommendations less frequently due to problems with understanding given advice and putting it into practice, which may lead to a vicious circle.

From a neurobiological perspective, several interesting hypotheses have been put forward which might explain the frequent co-occurrence of psychiatric symptoms in patients with asthma or COPD: systemic inflammation seems to be a key link between these conditions and studies conducted so far suggesting that cytokines might have an indirect effect on the neurotransmission in the central nervous system (CNS).

Cytokines comprise a heterogeneous group of polypeptide mediators of intercellular communication that are classically associated with activation of the immune system. Cytokines and their receptors are expressed in tissues of the nervous system (Hopkins and Rothwell 1995). Cytokine signaling in the brain is known to regulate important brain functions (neurotransmitter metabolism, neuroendocrine function, synaptic plasticity, neural circuitry of mood). Therefore, it is not surprising that a dysregulation in cytokine signaling might lead to occurrence of depression, anxiety and cognitive dysfunction (Salim et al. 2012). The effect of interleukin 6 (IL-6) has been most exhaustively described (Służewska et al. 1995; Alesci et al. 2005; O’Donovan et al. 2010) but among the proinflammatory mediators investigated so far are also interleukin-1 beta (IL-1β), tumor necrosis factor alpha (TNF-α) and C-reactive protein (CRP) (Ford and Erlinger 2004; Hashmi et al. 2013; Lichtblau et al. 2013). The excess or prolonged production of proinflammatory cytokines can lead to chronic inflammation and disorders like anxiety or depression (Maes 1999; Raison et al. 2006; Krishnadas and Cavanagh 2012).

Furthermore, frequently administered medications such as corticosteroids and H1-blockers also have an impact on a patient’s mental condition (Brown et al. 2007; Brown 2009). They cause a deficit of attention and psychomotor retardation, making social functioning difficult and worsening the quality of life.

Finally, obstructive pulmonary diseases as chronic and untreatable conditions may lead to depression and anxiety due to the high stress levels and general worries they cause in patients.

Apart from their impact on individual patients, asthma and COPD also constitute a major public health problem. The prevalence of asthma, according to GINA (Global Initiative for Asthma), is estimated to be around 300 million worldwide (Masoli et al. 2004). In spite of the fact that worldwide prevalence of COPD is lower (around 64 million), it imposes a heavier disease burden. According to The Global Burden of Diseases, Injuries, and Risk Factors Study 2010, COPD is the 3rd leading cause of global death and premature death. In addition, it is the 7th cause of DALY (disability adjusted life years) and 10th cause of YLL (years of life lost) (Institute for Health Metrics and Evaluation 2013).

The common denominator of both diseases is chronic inflammation leading to airway obstruction. The main difference is the distinct features of inflammation and reversibility of obstruction—it is reversible in asthma, while it progressively worsens and is irreversible in COPD. In asthma, the inflammatory infiltrate is predominantly composed of eosinophils, mast cells and CD4 T (Th2) lymphocytes, while in COPD it is mainly formed by neutrophils, macrophages and CD8 T (Th1) lymphocytes. This distinction is very important as the features of inflammation implicate the response to treatment—inhaled corticosteroids are effective against the eosinophilic inflammation in asthma, but largely ineffective against the primarily neutrophilic inflammation seen in COPD (Barnes et al. 2002).

Given these immunological changes typical for asthma and COPD, the above described hypotheses of neuroimmunological alterations also causing the psychiatric symptoms often observed in these patient groups is very intriguing. Therefore, the aim of this study was to reveal a possible correlation between immunological parameters such as cell counts in induced sputum and psychoemotional functioning as well as intellectual abilities, a research approach which has not been frequently used so far. The main risk factor of COPD—inhalation of tobacco smoke—is responsible for up to 90 % of COPD cases (Doherty and Briggs 2004). Also, up to 30 % patients with asthma are smokers (Gibson and Simpson 2009). Asthma patients with a history of smoking exhibit higher numbers of neutrophils in bronchoalveolar lavage fluid and biopsies (Wenzel et al. 1997). In contrast, COPD patients, who almost all have a history of smoking, usually exhibit eosinophilia during acute disease exacerbations (Saetta et al. 1994).

Apart from obvious similarities there are distinct differences both on the internal medicine/immunological as well as the psychosocial level which makes it worthwhile to look at both conditions separately. Therefore it’s worth comparing those two groups in terms of psychoemotional and cognitive functioning. Especially worth testing is a hypothesis that depressive symptoms and cognitive impairment are more common in COPD and anxiety symptoms are more common in asthma.

So far, as most studies focus on either severe or all stages of the disease, we decided to examine the under-research subgroup of patients with mild to moderate asthma or COPD. In addition, we aimed at quantifying the scores for depressive and anxiety symptoms as well as cognitive functioning in patients with these two pulmonological diseases using appropriate psychometric instruments and to compare them with those of healthy controls. This study is part of a wider ongoing research initiative. In our earlier work on a smaller group of patients, we observed that the level of anxiety and depression in asthma and COPD patients measured by self-assessment questionnaires was higher than in the control group. Also cognitive functions were worse than in the healthy controls, especially in COPD patients (Bratek et al. 2013).

## Subjects and methods

### Subjects

The participants of the study were recruited among patients of the Department of Pulmonology, Central Clinical Hospital of Medical University of Silesia. We recruited 59 people, of whom 24 were diagnosed with COPD and 17 with asthma. 18 individuals without lung diseases were included as controls. All participants signed an informed consent prior to taking part in the study. The research protocol was approved by the Ethics Committee of the Medical University of Silesia. In the asthma group there were 17 people (8 women and 9 men), mean age 51 ± 20 who met the GINA (The Global Initiative for Asthma) criteria for asthma^1^ and were in a stable period of the disease (based on medical history and physical examination) (Table 1).

**Table 1:** Baseline characteristics of the study population

	Asthma group (*n* = 17)	COPD group (*n* = 24)	Control group (*n* = 18)
Age (years)	51 ± 20	67 ± 7	48 ± 14
Females (%)	47 % (*n* = 8)	33 % (*n* = 8)	83 % (*n* = 15)
Males (%)	53 % (*n* = 9)	67 % (*n* = 16)	17 % (*n* = 3)
Smokers (%)	47 % (*n* = 8)	96 % (*n* = 23)	44 % (*n* = 8)
Pack-years (mean ± SD)	8 ± 14	22 ± 14	8 ± 12
FEV1 (% predicted) (mean ± SD)	78 ± 29	60 ± 20	100 ± 16
FEV1/FVC ratio (mean ± SD)	81 ± 34	57 ± 24	97 ± 10

Values are expressed as the mean ± SD or percent. Values were rounded to whole numbers

*COPD* chronic obstructive pulmonary disease; *FEV1* forced expiratory volume in 1 s; *FVC* forced vital capacity; *SD* standard deviation

The COPD group consisted of 24 people (8 women and 16 men) with a mean age 67 ± 7. Inclusion criteria for this group were: typical symptoms as assessed by a standardized interview (exertional dyspnea, progressive, chronic, productive cough), FEV1/FVC < 0.7 after inhalation of bronchodilators and stable period of the disease (on the basis of medical history and physical examination) (Table 1).

The control group consisted of 18 people (15 women, 3 men), mean age 48 ± 14, who did not suffer from other chronic diseases of the respiratory system, and did not report and were never treated for any mental health disorder (Table 1). Unfortunately, it was not possible to find a sufficient number of male volunteers who were willing to participate in this study. Therefore, the gender distribution between patient and control groups differed considerably.

Exclusion criteria for all three groups were: no voluntary written informed consent to participate in the study, poorly controlled asthma with frequent exacerbations, COPD in stage III and IV according to Global Initiative for Chronic Obstructive Lung Disease (2013), contraindications to perform spirometry (aneurysms of the aorta or cerebral arteries, history of retinal detachment or recent ophthalmic surgery, hemoptysis of unknown origin, pneumothorax, myocardial infarction or stroke during hospitalization, the state after surgery of the abdomen or chest), pregnancy, exacerbation of the disease currently or within the last 4 weeks, validated cognitive deficits disabling participation in the study (based on MMSE), psychiatric disorders requiring constant therapy and organic primary CNS damage.

### Instruments

Mini-Mental State Examination is a brief questionnaire test that is used to screen for cognitive dysfunction. In our study, it was used to exclude patients with cognitive impairment (<25 points).

The Trail Making Test (TMT) Part A and B measures attention, speed, mental flexibility, spatial organization, visual pursuits, recall and recognition (Corrigan and Hinkeldey 1987). Both parts of the test consist of 25 circles printed on a sheet of paper. The patient’s task is to connect the numbers (Part A) or numbers and letters (Part B) in ascending order. Some deficiency can be presumed when the time required by a patient to complete the task is longer than 78 s for Part A and 273 s for Part B.

Beck Depression Inventory (BDI) is a self-assessment questionnaire developed in 1961 and widely used for measuring the severity of depression (Beck et al. 1961). In this study, a cut-off of 12 points was chosen according to previously published studies of Polish populations (Wojnar et al. 2003; Dróżdż et al. 2007; Dudek et al. 2007).

State-Trait Anxiety Inventory for Adults is a self-report questionnaire to measure anxiety (Speilberger and Sarason 1975). It consists of two parts: Part 1 measures state anxiety (anxiety about an event) and Part 2—trait anxiety (anxiety level as a personal characteristic). Higher scores are positively correlated with higher levels of anxiety.

### Procedures

After the clinical interview and physical examination, all the participants completed psychometric tests and underwent spirometry and sputum induction. All the procedures were performed within the same day.

All psychometric tests were performed in the Department of Pulmonology, Central Clinical Hospital of Medical University of Silesia. In this naturalistic pilot study, the psychometric instruments were administered by the clinicians responsible for the patients’ treatment. Therefore, the raters were not blind towards the pulmonological diagnosis of the participants.

FEV1 (forced expiratory volume in 1 s) is the most frequently used spirometric index for assessing airway obstruction. The lower the FEV1, the greater the severity of the disease (MacNee et al. 2009).

Sputum induction is a useful method that involves inhalation of isotonic or hypertonic solutions (in our study sodium chloride) administered by nebulization. It allows to induce a certain amount of sputum that can be expectorated and analyzed to study the features of airway inflammation in respiratory disorders (Paggiaro et al. 2002). As data available on this subject is limited, we performed a statistical analysis of correlation of all measured factors and the percentage of particular cells in induced sputum with the use of Spearman’s rank correlation coefficient. In all performed tests, a higher score indicated a greater impairment in the measured factors; therefore, a positive correlation was related to worse results on the performed test. Accordingly, negative correlation was connected with better psychoemotional and cognitive functioning. Spirometry and sputum induction were performed in Pulmonary Function Testing Laboratory at Central Clinical Hospital in Katowice.

### Statistical analysis

Statistical analysis was conducted with Statistica v. 12 Software. Continuous variables were compared using the Mann–Whitney *U* test. Categorical variables were compared by Chi square test. Correlations were evaluated using Spearman’s rank correlation coefficient. A *p* value of <0.05 was considered significant. The results were rounded to whole numbers.

## Results

All neuropsychological tests indicating depressive, anxiety and cognitive symptoms were negatively correlated with the relative amount (percentage) of macrophages which is typical for inflammatory infiltrate in COPD and is detected via sputum induction. The correlation was statistically significant for TMT A and B scores. The relative amount (percentage) of neutrophils (also typical for COPD) correlated positively as well with all neuropsychological scores with a statistically significant correlation for TMT A score. The percentage of eosinophils (typical for inflammatory infiltrate in asthma) was weakly negatively correlated with MMSE, STAI and TMT A, but not with TMT B and BDI. The percentage of lymphocytes (may be increased in both asthma and COPD) was weakly negatively correlated with all the scores except for TMT B and STAI-1 (Table 3).

FEV1 was negatively correlated with all scores, i.e. more severe airway obstruction was associated with worse psychoemotional and cognitive functioning. This correlation was statistically significant for TMT A (Table 3).

Regarding the group results of the individual neuropsychiatric tests, the mean MMSE score was 29 ± 1 point. The lowest score (26) was found in a patient of the COPD group (Table 2).

**Table 2:** Comparison of cognitive functions, depression and anxiety status of the study population

	TMT A (s)	TMT B (s)	BDI (points)	STAI-1 (points)	STAI-2 (points)	MMSE (points)
Asthma group	35 ± 17	96 ± 43	8 ± 7	35 ± 11	40 ± 11	29 ± 1
COPD group	42 ± 17*	107 ± 41*	11 ± 7	38 ± 9*	42 ± 7	28 ± 1
Control group	35 ± 11	70 ± 23	7 ± 7	34 ± 9	37 ± 6	29 ± 1

Values are expressed as the mean ± SD (standard deviation). Group differences are significant at *p* < 0.05. A *p* < 0.05 compared to healthy controls is marked with an asterisk

*COPD* chronic obstructive pulmonary disease; *TMT* trail making test; *BDI* beck depression inventory; *STAI* state-trait anxiety inventory; *MMSE* mini-mental state examination

Regarding the mean time in the TMT, the control group exhibited the best results (Part A: 35 ± 11 s, Part B: 70 ± 23 s), second best was the asthma group (Part A: 35 s ± 17 s, Part B: 96 ± 43 s) and least was the COPD group (Part A: 42 ± 17 s, Part B: 107 ± 41 s) (Table 2). The difference between COPD group and control group was statistically significant (*p* = 0,011 in TMT Part A and *p* = 0,002 in TMT Part B). In Part A, two patients (one with asthma and one with COPD) were above the threshold for possible cognitive dysfunctions of clinical relevance. We also found a significant correlation between TMT score and age, pack-years, FEV1 (only in Part A), the percentage of macrophages and the percentage of neutrophils (only in Part A) (Table 3).

**Table 3:** Correlation of psychoemotional and cognitive functions with: induced sputum cellularity, FEV1, pack-years and age

	TMT A	TMT B	BDI	STAI -1	STAI-2
Macrophages (%)	−0.319952*	−0.353752*	−0.195457	−0.052123	−0.089541
Neutrophils (%)	0.294920*	0.154875	0.193042	0.114351	0.113984
Eosinophils (%)	−0.024736	0.211875	0.001979	−0.140019	−0.027906
Lymphocytes (%)	−0.006706	0.007992	−0.148744	0.026009	−0.023340
FEV1 (%)	−0.350425*	−0.262999	−0.169495	−0.193690	−0.181556
Pack-years (years)	0.332659*	0.381675*	0.378157*	0.342069*	0.353231*
Age (years)	0.480223*	0.572703*	0.401527*	0.231369	0.376620*

Correlation between the percent of macrophages, neutrophils, eosinophils, lymphocytes; FEV1/FVC; pack-years; age and the results of *TMT* trail making test, *BDI* beck depression inventory and *STAI* state-trait anxiety inventory. Statistical analysis was performed with Spearman’s rank correlation coefficient. Correlations marked with an asterisk are significant at *p* < 0.05

The lowest mean BDI score was obtained in the control group (7 ± 7), the asthma group was in the middle (mean 8 ± 7) and highest scores were found in the COPD group (mean 11 ± 7) (Table 2). The difference between the COPD group and control group was statistically significant (*p* = 0,019). A score higher than 12 (but lower than 33) was found in 14 patients: 8 of COPD group (33 % of all COPD patients), 5 of asthma group (29 % of all asthma patients) and in 1 healthy control (0.05 % of the control group). There was no statistically significant gender difference in the BDI scores, scores higher than 12 were found in 29 % of the women and 21 % of the men. Also, age and pack-years were positively correlated with BDI (Table 3).

The STAI scores within the groups were as follows: in Part 1 the mean score of the asthma group was 35 ± 11, of the COPD group 38 ± 9 and of the control group 34 ± 9. The difference between the COPD group and the control group was statistically significant (Table 2).

As for the Part 2, the mean score in the asthma group was 40 ± 11, in the COPD group 42 ± 7 and in the control group 37 ± 6 (Table 2). There was no correlation between age and the STAI score. A threshold score of 44 was chosen which is considered the lowest STAI threshold that can be used for the detection of a possible anxiety disorder (Antony et al. 2001). Scores higher than or equal to 44 on one part or both parts of the STAI were observed in 20 participants, among them 7 asthma patients (41 %), 10 COPD patients (42 %) and 3 individuals of the control group (17 %). There was no gender-related statistically significant difference in the STAI scores; scores higher than or equal to 44 points were found in 39 % of the women and in 32 % of all men. Interestingly, there was a significant correlation between STAI, age (only in Part 2) and pack-years (Table 3).

## Discussion

We were able to demonstrate a correlation of the neuropsychological test results measuring depressive, anxiety and cognitive symptoms of asthma and COPD patients with some immunological/inflammatory (relative amount of leukocytes using sputum induction) and pulmonological (FEV1) key parameters.

In addition, the results of our study indicated that scores indicative for the presence of psychiatric symptoms were higher in pumonological patients than controls, similar to previous published work (Brenes 2003; Hasler et al. 2005; Lavoie et al. 2006; Carvalho et al. 2007; Goodwin et al. 2010; Wang et al. 2010; Asnaashari et al. 2012).

These findings are of especial clinical relevance because psychiatric comorbidities, although relatively common among asthma and COPD patients, remain often undetected and untreated in daily clinical practice. This might be due to the fact that on one hand the screening tools for depression and anxiety disorders are not routinely employed and that on the other hand patients often tend to deny that they are suffering from psychiatric conditions due to the stigma attached to them (Yohannes et al. 2000).

Nevertheless, our findings are largely in line with the published data of other groups that the severity of pulmonary obstruction is a risk factor for and correlates strongly with both anxiety and depression levels (Kühl et al. 2008; Omachi et al. 2009; Balcells et al. 2010; Martínez-Rivera et al. 2011; Di Marco et al. 2006). In the ELIPSE study prevalence of depression was higher as COPD became more severe, although scores were more closely related to health status and symptoms, than to lung function (Hanania et al. 2011). There are also studies indicating the correlation between dyspnea, anxiety-trait and anxiety-state, neuroticism and depression in asthmatics (Nowobilski et al. 2007). According to systematic reviews, the prevalence of clinical depression in these patients ranges between 7 and 42 % (van Ede et al. 1999), while that of anxiety ranges between 10 and 19 % (Maurer et al. 2008). Already in the 80 s, Yellowlees et al. (1987) studied 50 patients with COPD and—using the DSM-III criteria—found that 34 % of the patients also suffered from an anxiety disorder and 16 % from clinical depression. In contrast to these findings, some study results have been published indicating even higher prevalence rates of depression in COPD patients. Kunik et al. (2005) screened the patients with the chronic breathing disorders (including COPD) with the primary care evaluation of mental disorders (PRIME-MD). 80 % screened positive for depression, anxiety or both. In a recent Iranian study, the prevalence of depression (estimated with Beck Depression Inventory) in COPD group was 54.2 % compared to 66.7 % in asthmatic group and 44.4 % in asthmatic bronchitis groups (Asnaashari et al. 2012). The depression scores were directly related to severity of pulmonary obstruction. Using the Hospital Anxiety and Depression Scale (HADS) at hospital admission, Dowson et al. (2001) found anxiety in 50 % and depression in 28 % of 72 patients with COPD hospitalized for cardiorespiratory rehabilitation.

However, it is note-worthy that our study did not aim at establishing the prevalence of psychiatric disorders such as depression and anxiety disorder in pulmonological patient groups, as was done in the above-mentioned studies, but rather to correlate the results of neuropsychological tests with the results of pulmonological, i.e. biological examinations.

In the ELIPSE study, it was observed that 26 % of COPD patients were depressed compared to 12 % of non-COPD smokers and 7 % of non-COPD non-smokers. Depression was measured with The Center for Epidemiological Studies-Depression Scale (CES-D) (Hanania et al. 2011). There are findings suggesting that the association between anxiety, depression and COPD may be at least partly explained by cigarette smoking and nicotine dependence (Goodwin et al. 2012).

Those findings are corroborated by our study in which pack-years correlated positively with all neuropsychological factors examined on a statistically significant level which means that the long-term exposure to tobacco smoke is related to a deterioration of cognitive functions and higher level of depressiveness and anxiety.

Gender is another potential risk factor affecting patient’s situation, but different studies reported inconsistent results. An international WHO survey reported that age and gender adjusted odds ratios for depressive and anxiety disorders in asthmatic patients are 1.6 and 1.5, respectively (Scott et al. 2007). In Wilson’s study, asthmatic men and women had similar prevalence of psychological morbidities (Wilson et al. 2010).

This is in line with the results of our study which did not show statistically significant gender-related differences. Nevertheless, it remains a shortcoming of our study that the control group consisted predominantly of women. Nowobilski et al. (2011) stated that asthmatic women experience higher degrees of somatic symptoms and anxiety than men. Also, the women had higher values for depression, anxiety-trait and neuroticism (Nowobilski et al. 2006). In the Asnaashari study, females suffering from obstructive lung diseases had a worse psychological status compared to male patients (Asnaashari et al. 2012).

Cognitive dysfunction is highly prevalent in both COPD and asthma patients (Schou et al. 2012; Klein et al. 2010). In a study including only non-hypoxemic patients with stable COPD they performed significantly worse on TMT B, the digit-symbol test and on the addition subtest (Liesker et al. 2004). In a study that enrolled COPD patients with mild hypoxemia they performed significantly worse than controls on verbal fluency tasks, however, they were not in the clinically impaired range (Kozora et al. 1999). The first study providing information on cognitive dysfunctions in asthma patients was conducted in 1981 and revealed that a majority of the asthmatics manifested definite signs of memory impairment when they were asked to recall (Schraa et al. 1981). In the largest sample cross-sectional study so far asthma was associated with 78 % increased risk of cognitive impairment when controlling for demographic characteristics, self-rated health status, inhaled corticosteroid use and FEV1/FVC (Caldera-Alvarado et al. 2013).

For measuring cognitive functions in our study we used TMT A and B. We found that the time required to complete the task was significantly longer in patients with COPD. We also found a significant positive correlation between TMT score (higher scores indicate worse cognitive function) and the age and pack-years (Table 3).

Further researches based on such neuroimmunological hypotheses are urgently needed on the clinical level, but also on the translational level of animal models, to reveal the fundamental neuropathological mechanisms underlying the increased prevalence of psychiatric symptoms in asthma and COPD patients described in epidemiological studies.

## Conclusions

According to the results of our study, patients with mild to moderate asthma and COPD exhibit higher levels of depressiveness, anxiety symptoms and cognitive dysfunction than healthy controls; however, in very mild cases, this phenomenon is statistically not significant. This is also reflected in the finding that the severity of airflow obstruction is correlated with the level of all measured neuropsychological factors which means that the deterioration of cognitive functions and levels of anxiety and depressive symptoms become even more severe as the pulmonological disease progresses. The amount of exposure to tobacco smoke was also found to be strongly correlated with worse psychoemotional state and cognitive function. Thus, one conclusion should be that psychiatric care should be an integral part of the treatment plans for those patients and that especially treating nicotine dependence might help to improve not only their lung problems but also their neuropsychological condition and thereby quality of life.

## Footnotes

1 typical symptoms (recurring episodes of wheezing, breathlessness, chest tightness and coughing, particularly at night or in the early morning), variable and reversible airway obstruction on spirometry tests—recently or documented in the past.
